# Identification of key modules and hub genes for sepsis-induced myopathy using weighted gene co-expression network analysis

**DOI:** 10.3389/fgene.2025.1607575

**Published:** 2025-07-28

**Authors:** Siming Lin, Kexin Cai, Ai Chen, Weibin Wu, Guili Lian, Shaodan Feng, Zhihong Lin, Liangdi Xie

**Affiliations:** ^1^ Department of Emergency, The First Affiliated Hospital, Fujian Medical University, Fuzhou, China; ^2^ Department of Emergency, National Regional Medical Center, Binhai Campus of the First Affiliated Hospital, Fujian Medical University, Fuzhou, China; ^3^ Fujian Hypertension Research Institute, The First Affiliated Hospital of Fujian Medical University, Fuzhou, China; ^4^ Clinical Research Center for Geriatric Hypertension Disease of Fujian Province, The First Affiliated Hospital of Fujian Medical University, Fuzhou, China; ^5^ Department of Geriatrics, The First Affiliated Hospital of Fujian Medical University, Fuzhou, China; ^6^ Branch of National Clinical Research Center for Aging and Medicine, The First Affiliated Hospital of Fujian Medical University, Fuzhou, China; ^7^ Department of Geriatrics, National Regional Medical Center, Binhai Campus of the First Affiliated Hospital, Fujian Medical University, Fuzhou, China

**Keywords:** sepsis-induced myopathy, skeletal muscle, hub genes, biomarkers, bioinformatics, WGCNA

## Abstract

**Background:**

Sepsis-induced myopathy (SIM) is a severe complication of sepsis, leading to significant muscle dysfunction and increased mortality. The molecular mechanisms underlying SIM remain poorly understood, necessitating comprehensive studies to identify potential therapeutic targets. This study aims to explore the molecular basis of SIM through gene expression analysis and bioinformatics approaches.

**Methods:**

In this study, we employed a lipopolysaccharide-induced mouse model to investigate the molecular basis of SIM. We conducted comprehensive RNA sequencing of the gastrocnemius muscle, which resulted in the identification of 1,166 genes exhibiting altered expression levels. To further analyze the data, we applied weighted gene co-expression network analysis (WGCNA) to distinguish critical gene clusters associated with SIM. Additionally, we performed functional enrichment analyses using Gene Ontology (GO), Kyoto Encyclopedia of Genes and Genomes (KEGG), and protein-protein interaction (PPI) network approaches.

**Results:**

Our findings revealed that the identified gene clusters predominantly pertained to immune response, inflammation, and apoptosis pathways. Notably, validation through real-time quantitative polymerase chain reaction (RT-qPCR) confirmed the significant upregulation of key hub genes, including Cxcl10, Il6, and Stat1. Receiver Operating Characteristic (ROC) curve analysis further indicated the potential diagnostic utility of these hub genes. Additionally, leveraging the Connectivity Map (CMAP) database allowed us to predict six potential pharmacological agents—halcinonide, lomitapide, TG-101348, GSK-690693, loteprednol, and indacaterol—that might serve as therapeutic interventions for SIM.

**Conclusion:**

This research advances our understanding of the molecular basis of SIM, presenting new diagnostic biomarkers and potential drug targets. Further studies with larger clinical datasets are warranted to validate these findings and explore the therapeutic potential of the identified drugs.

## 1 Introduction

Sepsis-induced myopathy (SIM) is an acquired muscle-wasting disease caused by sepsis (Wollersheim et al., 2014). It primarily involves acute and progressive atrophy of the skeletal and respiratory muscles, leading to prolonged mechanical ventilation duration, extended intensive care unit (ICU) stays, increased medical costs, long-term functional disabilities, and higher patient mortality rates (Hermans et al., 2014; Friedrich et al., 2015; Hermans and Van den Berghe, 2015). Current treatment strategies for sepsis, including antibiotics and supportive care, have limited efficacy in preventing or reversing muscle wasting and weakness associated with SIM (Singer et al., 2016; Yoshihara et al., 2023). Hence, it is crucial to clarify the molecular pathways involved in SIM and discover possible treatment targets to enhance patient results.

The pathophysiology of SIM is complex and multifactorial, involving inflammation, oxidative stress, mitochondrial dysfunction, and altered protein metabolism (Lang et al., 2007; Fredriksson et al., 2008; Callahan and Supinski, 2009; Powers et al., 2012; Singer, 2014). Prior research has pinpointed various essential molecular pathways associated with muscle wasting and impairment in other severe illness-related muscle disorders, including critical illness polyneuropathy and intensive care unit-acquired weakness (Hermans and Van den Berghe, 2015). During critical illness, muscle protein degradation is known to be influenced by the ubiquitin-proteasome system and autophagy-lysosome pathway, which play important roles (Schakman et al., 2013). Moreover, inflammatory cytokines like IL-6 and tumour necrosis factor-α (TNF-α) have been demonstrated to play a role in muscle loss by encouraging the breakdown of proteins and hindering the creation of muscle proteins (Argilés et al., 2005; Pedersen and Febbraio, 2008). However, the specific molecular mechanisms and gene expression changes associated with SIM remain poorly understood, highlighting the need for comprehensive studies to identify novel biomarkers and therapeutic targets.

Our goal in this research was to explore the molecular pathways of SIM by utilizing a mouse model of sepsis triggered by the injection of lipopolysaccharide (LPS). RNA-seq was conducted on gastrocnemius muscle samples from mice with sepsis and a control group to detect genes that were expressed differently. WGCNA was used to identify key gene modules associated with SIM. Analyses were performed to investigate the biological functions and pathways enriched in the identified DEGs, including GO and KEGG pathway analyses. PPI networks were built to discover hub genes, which were then confirmed through RT-qPCR analysis. We used the CMAP database to forecast potential small molecule medications for treating SIM.

Our study identified several key DEGs and gene modules associated with SIM, providing new insights into the molecular mechanisms underlying this condition. Functional enrichment analyses showed that the DEGs participate in a range of biological processes such as immune response, inflammation, and muscle protein metabolism. PPI network analysis identified several hub genes, including Cxcl10, Il6, and Stat1, which were further validated by RT-qPCR. ROC curve analysis demonstrated that these hub genes have high diagnostic potential for SIM. Furthermore, CMAP analysis predicted several small molecule drugs that may have therapeutic potential for reversing SIM. Our findings provide a valuable resource for understanding the molecular basis of SIM and highlight potential therapeutic targets for this debilitating condition.

## 2 Methods

### 2.1 Experimental design for animal studies

The purpose of this research was to analyze the alterations in skeletal muscle gene expression during the initial stages of sepsis by conducting RNA sequencing on samples from the gastrocnemius muscle of mice with early sepsis and comparing them to samples from control mice. This investigation aimed to uncover the potential molecular pathways involved in sepsis-induced muscle wasting. Sixteen male C57/BL6J mice, in good health and weighing around 25 g, were acquired from Shanghai SLACCAS Laboratory Animal Co., Ltd. (quality certificate SCXK 2017-0005). The mice were all bred in a regulated animal facility with a consistent temperature of 22°C ± 2°C, humidity of 55% ± 5%, and a 12-h light-dark cycle. They were given free access to food and water. Following a week of adjustment, the sixteen mice were split into two groups at random: the sepsis group was given an intraperitoneal injection of LPS (10 mg/kg), while the control group received the same amount of 0.9% saline. LPS was used to induce a sepsis-like systemic inflammatory response, particularly reflecting Gram-negative bacterial infection (Lang et al., 2007; Callahan and Supinski, 2009). The selected dosage of 10 mg/kg was based on previous reports (Zeng et al., 2024) demonstrating that this dose induces robust systemic inflammation and early skeletal muscle catabolism within 18–24 h, without excessive mortality in healthy C57BL/6J mice. The gastrocnemius muscle was harvested for RNA-seq analysis due to its well-defined anatomy, availability of sufficient tissue, and prior validation in sepsis-induced myopathy studies (Lang et al., 2007). The LPS utilized in the research was acquired from Sigma (China) and originated from *Escherichia coli* (055: B5). Referencing the previous research by Kim, J’s team (Kim et al., 2020), the mice were humanely euthanized within 18 h post-injection. Their gastrocnemius muscles were quickly collected, rapidly frozen in liquid nitrogen, and stored at −80°C. After gathering gastrocnemius tissue from all groups, eight samples from the sepsis group and seven samples from the control group were chosen for RNA sequencing. The sample size was based on prior study using RNA-seq to analyze gene expression in skeletal muscle following LPS administration, where group sizes of 6-8 have been shown sufficient to detect significant transcriptional changes (Lang et al., 2007). The number of animals was also determined in accordance with the 3Rs principle to minimize animal usage while ensuring data robustness. Approval for this research was granted by the Fujian Medical University Laboratory Animal Welfare and Ethics Committee under the reference number IACUC FJMU 2022-NSFC-0181, with all protocols following ethical guidelines. Procedures were conducted using sodium pentobarbital as an anesthetic, with the goal of reducing pain.

### 2.2 RNA extraction and sequencing

Extraction of RNA was performed with the Rneasy mini plus kit from Qiagen. A total of 1.3 μg of RNA was used for constructing sequencing libraries. The RNA-seq libraries were created with the Qiagen mRNA-Seq library Prep Kit according to the manufacturer’s instructions. SOAPnuke (v1.5.6) was utilized to filter the original sequencing data by eliminating reads with junctions, unknown base N content exceeding 5%, and low-quality reads (defined as reads with a ratio of bases with quality value below 15 to the total bases in the read surpassing 20%). The clean data that was obtained was subsequently analyzed, mapped, and processed with the assistance of the Dr. Tom Multi-Organomics Data Mining System available at https//biosys.bgi.com. The tab-delimited text format processed data files contain reads per kilobase million (RPKM) values for each sample.

### 2.3 Sequencing data upload and validation data download

The sequencing data has been submitted to the Gene Expression Omnibus (GEO) (Barrett et al., 2013) database at https://www.ncbi.nlm.nih.gov/geo. Processed data files are now accessible in tab-delimited text format, showing RPKM values for every sample. The GEO accession number for our data is GSE249141. The dataset GSE209706 related to sepsis-induced myopathy, which was obtained from the GEO database, was used in this study. The data platform used was GPL32276, which contained 9 samples derived from myoblast cells (3 saline control replicates, 3 LPS treated replicates, and 3 LPS + Moringa isothiocyanate-1 treated replicates). The samples from the control group and the LPS group were extracted as a validation set to validate the true hub genes in the *in vitro* models.

### 2.4 Identification of DEGs

DESeq2 package was utilized to conduct differential gene analysis, with a false discovery rate (FDR) < 0.05 and a |log2 fold change| > 2.0 as the thresholds for identifying DEGs in the comparison between sepsis and control samples. DEGs were displayed through volcano plots produced with the ggplot2 tool in R, along with a heatmap generated by the pheatmap package in R to exhibit the top 50 differentially upregulated and downregulated genes. Ultimately, we employed the tool “VennDiagram” to detect any common genes between key module genes and DEGs.

### 2.5 WGCNA analysis

To discover important modules and hub genes in early SIM, we utilized WGCNA to create a co-expression network with all gene expression data, following the guidelines for scale-free topology. At first, we examined the data with the WGCNA tool within the R program. A power of 9 was selected for soft-thresholding to create a network that is scale-free. Clusters containing a minimum of 30 genes were discovered using hierarchical clustering and the dynamic tree cut method, with modules being combined when their eigengene correlation surpassed 0.25. To achieve a balance between sensitivity and specificity in detection, the sensitivity parameter was set to 3. Subsequently, the association between each module and sepsis or control conditions was investigated. The modules that exhibited the strongest positive and negative correlations with sepsis were designated as key modules.

### 2.6 Functional enrichment analysis

We used the clusterProfiler package in R to analyze GO functions and KEGG pathways to identify the probable roles of the DEGs in the two key modules. The analysis of GO included molecular function (MF), cellular components (CC), and biological processes (BP). For gene enrichment analysis, a significance cut-off value of p. adjust <0.05 was set.

### 2.7 Identification of hub genes in the PPI network

PPI networks for DEGs in the two key modules were built using the STRING (Szklarczyk et al., 2023) website at http://string.embl.de/. Using CytoHubba, a plug-in for Cytoscape version 3.10.0, we identified the top 10 hub genes from the DEGs in the two key modules using nine algorithms (Betweenness, BottleNeck, Closeness, Degree, EcCentricity, EPC, MNC, Radiality, and Stress). Subsequently, the hub genes were visualized using the UpSetR package. The true hub genes were determined by identifying the genes that overlapped among the top 10 hub genes identified by nine different algorithms.

### 2.8 RT-qPCR of true hub genes

We selected the true hub genes, including Cxcl10, Il6, and Stat1, to validate their expression in mouse samples. RNA was isolated from the gastrocnemius muscle of sepsis (n = 6) and control groups (n = 6) using TRIzol reagent from Vazyme Biotechnology, China, following the manufacturer’s instructions. Following extraction, RNA from each sample was converted into cDNA through reverse transcription utilizing the Hifair®II 1st Strand cDNA Synthesis kit (YEASEN, China). Fluorescence quantification was performed using the qPCR SYBR^®^ Green Master Mix (No Rox) from YEASEN in China. Each reaction was conducted three times using a Light Cycler 96 system from Roche in Switzerland. GAPDH, a gene involved in maintaining the house, was used as a reference point. Gene expression levels were determined using the 2^−△△Ct^ technique. The primer sequences for all genes are shown in Table 1.

**TABLE 1:** Primer sequences for RT-qPCR.

Primer	Sequence (5′to3′)	Product size (bp)
CXCL10-F (mus)	CCG​CTG​CAA​CTG​CAT​CCA​TA	20
CXCL10-R (mus)	CAA​TGA​TCT​CAA​CAC​GTG​GGC	21
IL6-F (mus)	CTG​CAA​GAG​ACT​TCC​ATC​CAG	21
IL6-R (mus)	AGT​GGT​ATA​GAC​AGG​TCT​GTT​GG	23
STAT1-F (mus)	TGA​CGA​CCC​TAA​GCG​AAC​TG	20
STAT1-R (mus)	AGA​CAT​GGG​AAG​CAG​GTT​GT	20
GAPDH-F (mus)	TGG​AAA​GCT​GTG​GCG​TGA​TG	20
GAPDH-R (mus)	TAC​TTG​GCA​GGT​TTC​TCC​AGG	21

### 2.9 Examining ROC curves for true hub genes

A boxplot was created with the “ggplot2” package in R to show the difference in gene expression between sepsis and control samples. ROC analysis was performed with the “pROC” package in R to assess the diagnostic performance of the true hub genes, and the results were visualized using ggplot2. Genes exhibiting an area under the curve (AUC) exceeding 0.70 were noted to have the potential to offer diagnostic advantages for disease. The diagnostic efficacy of the true hub genes in the *in vitro* models was verified using GSE209706.

### 2.10 GeneMANIA analysis

The GeneMANIA (Warde-Farley et al., 2010) database, which focuses on building networks of protein-protein interactions, is available for access on the website http://www.genemania.org. The database illustrates the functional interconnections among genes and promotes exploration into their roles. The website offers a range of bioinformatics research techniques, such as physical interaction, gene enrichment analysis, gene co-localization, gene co-expression, and website prediction, along with the ability to choose gene node data sources. The central gene network for analyzing mechanisms was established using GeneMANIA.

### 2.11 Research on possible medications for treatment in the CMAP

For this study, we made use of the CMAP database available at https://clue.io/in order to forecast potential medications for treating the illness. CMAP (Subramanian et al., 2017) is a pharmacogenomics resource based on gene expression profiles that identifies potential drug inhibitors capable of reversing disease states by comparing gene expression changes induced by specific diseases and drug treatments. The study involved ranking potential medications according to connectivity scores and choosing the top six small molecule drug inhibitors as potential candidates. The PubChem (Kim et al., 2023) database was used to retrieve the 3D conformations of the leading six small molecule medications (https://pubchem.ncbi.nlm.nih.gov/).

### 2.12 Statistical analytics

The data was processed and analyzed with Excel from Microsoft and R software version 4.2.1. The independent Student’s t-test was utilized to assess statistical significance for normally distributed continuous variables, whereas the Mann-Whitney U test was employed for non-normally distributed variables in comparing two sets of continuous variables. Either the chi-square test or Fisher’s exact test was used to compare and analyze the statistical significance of two sets of categorical variables. The Kruskal–Wallis test was utilized to compare several groups, whereas the Wilcoxon test was utilized for comparing pairs of groups. Statistical significance was determined with a two-tailed p-value less than 0.05.

## 3 Results

### 3.1 Identifications of DEGs

**FIGURE 1:**
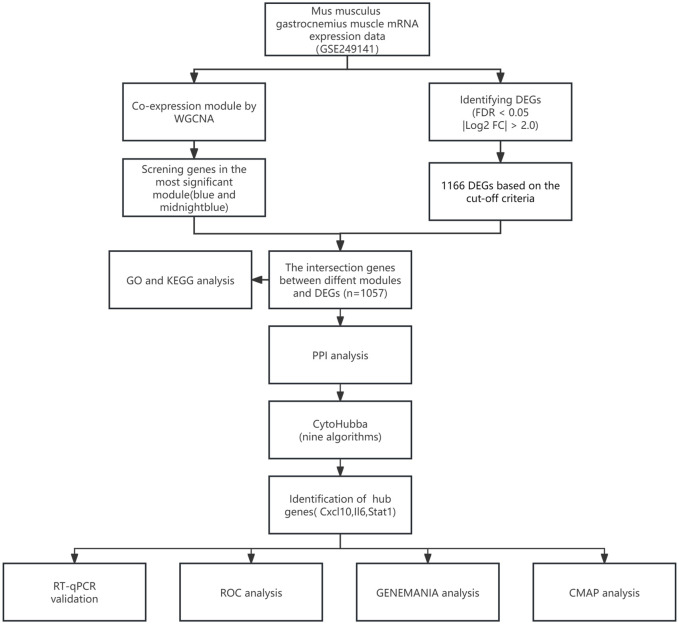
Study flowchart.

**FIGURE 2:**
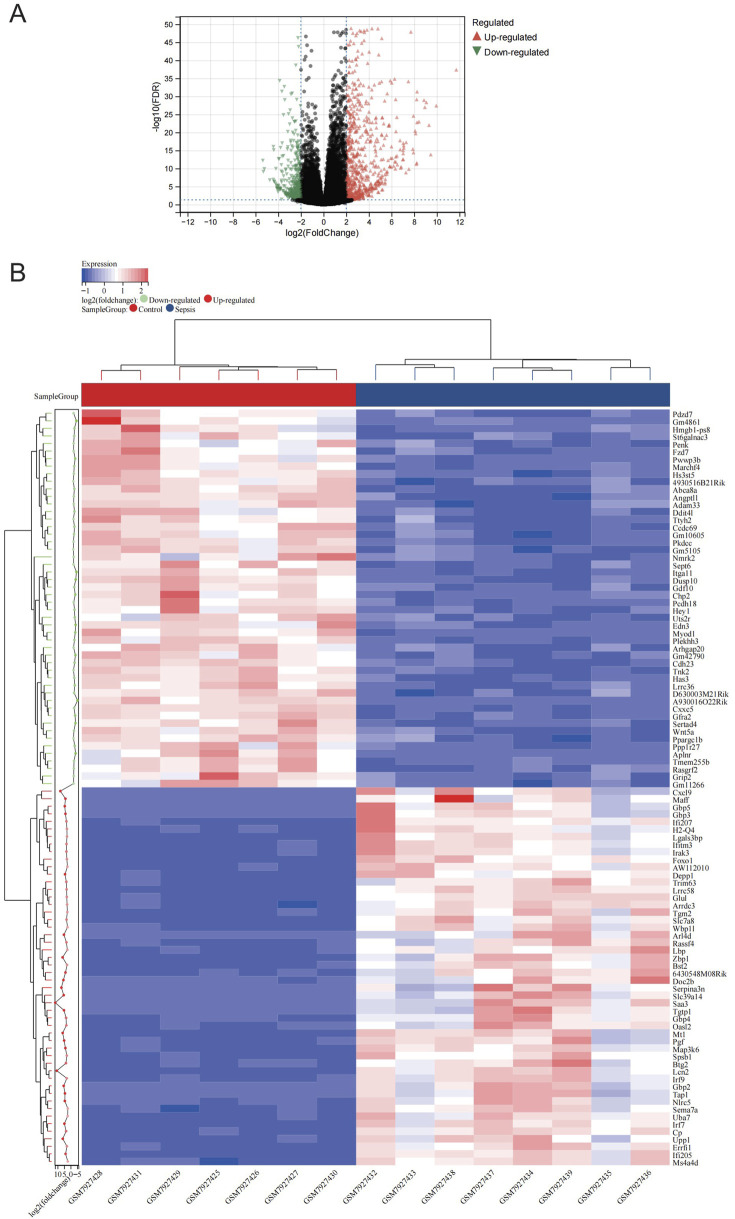
Expression profle of DEGs. **(A)** The map of volcanic DEGs. Black dots represent genes that are not diferentially expressed between sepsis and control. Downregulated genes are shown in green, while upregulated genes are shown in red. **(B)** Heatmap displaying the top 50 DEGs that are significantly upregulated and the top 50 that are significantly downregulated. Red signifies a higher gene expression value, while blue signifies a lower gene expression.

Figure 1 illustrates the study flow. A combined 1,166 DEGs were discovered, meeting the criteria of a FDR <0.05 and an absolute log2 fold change >2.0 when comparing the sepsis and control groups. Compared to the controls, there were 758 upregulated genes and 408 downregulated genes in sepsis. Figure 2A displays the volcano plot of DEGs. Figure 2B displays the heatmap of the top 50 differentially upregulated and downregulated genes.

### 3.2 WGCNA analysis

**FIGURE 3:**
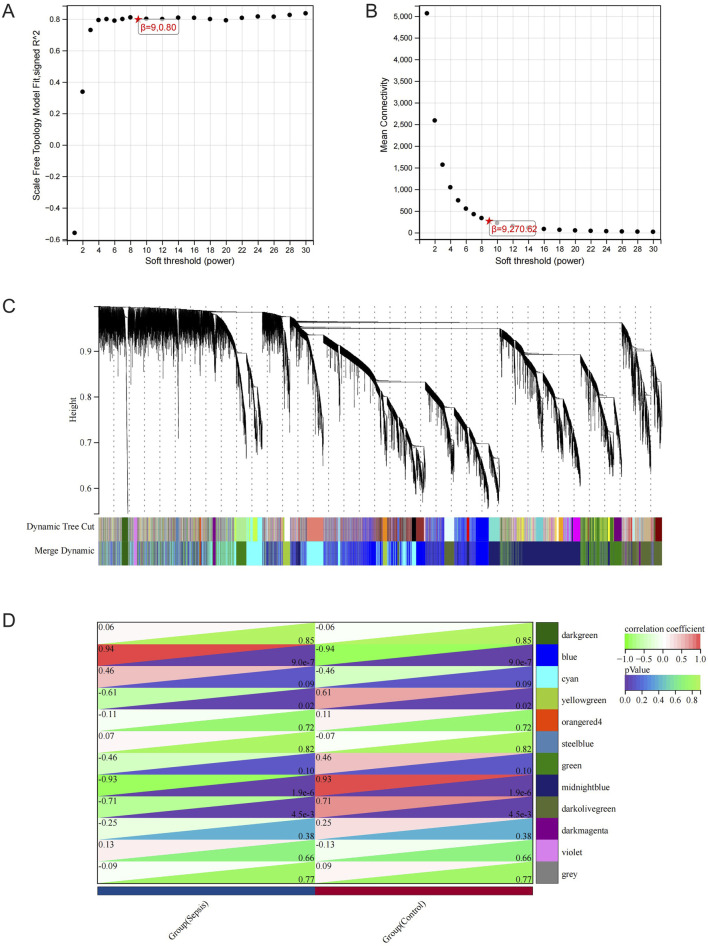
WGCNA Analysis. **(A)** Examining network structure for different soft-thresholding values. **(B)** Graph showing average connectivity based on different levels of soft-thresholding power. **(C)** Cluster dendrogram of gene modules. **(D)** Module-Trait relationships. Heatmap depicting the correlation between the module eigengenes and traits of sepsis and control conditions. The correlation and p-value are stored in each individual cell.

The RNA-seq dataset was used to process all gene expression data with the WGCNA package in R software. In the following analysis, the value of β for soft thresholding was determined to be 9 due to the achievement of a scale independence of 0.80 (Figures 3A,B) and maintained a fairly high level of connectivity on average. The genes were clustered into 12 modules: blue, cyan, darkgreen, darkmagenta, darkolivegreen, green, midnightblue, orangered4, steelblue, violet, yellowgreen, and grey, with a minimal module size >30. The cluster dendrogram of the genes is shown in Figure 3C. The association between every module and sepsis was computed and graphed (Figure 3D). The results indicated that blue (r = 0.94, p < 0.0001) was the most positively correlated module, and midnightblue (r = −0.93, p < 0.0001) was the most negatively correlated module related to sepsis. Thus, blue and midnightblue were designated as key modules.

### 3.3 Functional Enrichment Analysis

**FIGURE 4:**
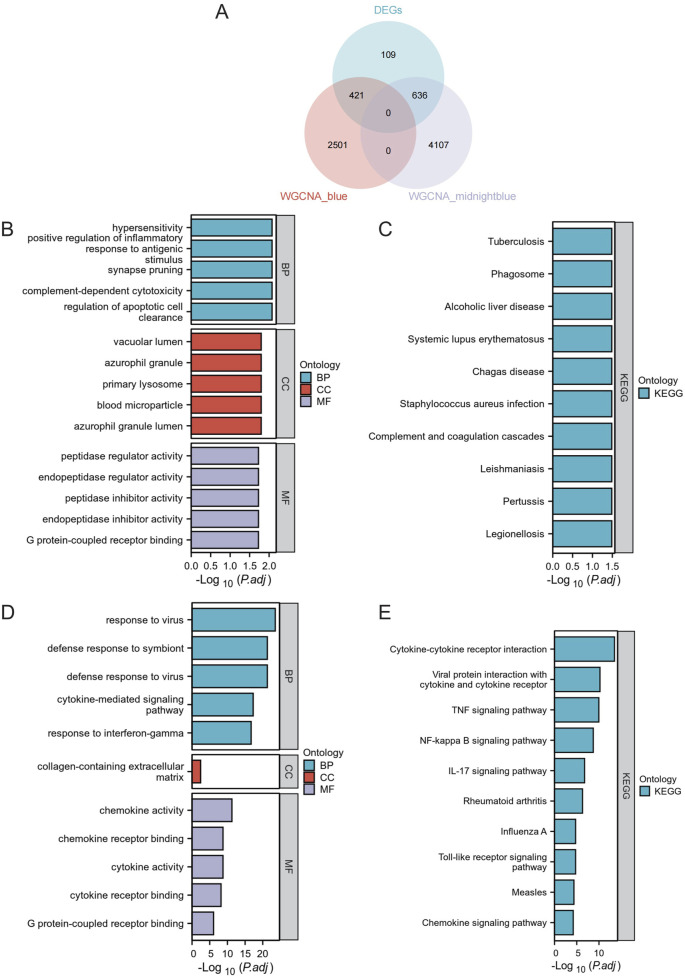
Functional Enrichment Analysis. **(A)** Venn Diagram of DEGs in Core Modules. Venn diagram illustrating the overlap of differentially expressed genes in the blue and midnightblue core modules. **(B)** GO enrichment analysis for blue module DEGs. **(C)** KEGG pathway analysis for blue module DEGs. **(D)** GO enrichment analysis for midnightblue module DEGs. **(E)** KEGG pathway analysis for midnightblue module DEGs.

To better analyze the overlap in key modules, the implicated genes were compared using a Venn diagram. Furthermore, 421 DEGs were classified as blue DEGs, while 636 DEGs were categorized as midnightblue DEGs (Figure 4A). The “clusterProfiler” program was used to analyze GO and KEGG enrichment in order to determine the most probable role of the DEGs in the two key modules. The GO analysis results indicated that there was a high presence of blue DEGs in hypersensitivity, promoting inflammation in response to antigenic stimulus, synapse pruning, complement-dependent cytotoxicity, regulating apoptotic cell clearance, vacuolar lumen, azurophil granule primary lysosome, blood microparticle, azurophil granule lumen, peptidase inhibitor activity, endopeptidase inhibitor activity, peptidase regulator activity, endopeptidase regulator activity, and G protein-coupled receptor binding (Figure 4B; Supplementary Table S1). The examination of KEGG revealed a strong correlation of these genes with tuberculosis, phagosome, alcoholic liver disease, systemic lupus erythematosus, chagas disease, *staphylococcus aureus* infection, complement and coagulation cascades, leishmaniasis, pertussis, legionellosis (Figure 4C; Supplementary Table S2). Furthermore, the GO terms were significantly overrepresented in midnightblue DEGs, such as virus response, symbiont defense response, virus defense response, signaling pathway mediated by cytokines, interferon-gamma response, extracellular matrix containing collagen, activity of chemokines, binding to chemokine receptors, activity of cytokines, binding to cytokine receptors, and binding to G protein-coupled receptors (Figure 4D; Supplementary Table S3). Shared genes enriched KEGG pathways included cytokine-cytokine receptor interaction, viral protein interaction with cytokine and cytokine receptor, TNF signaling pathway, NF-kappa B signaling pathway, IL-17 signaling pathway, rheumatoid arthritis, influenza A, Toll-like receptor signaling pathway, measles, and chemokine signaling pathway (Figure 4E; Supplementary Table S4).

### 3.4 PPI network analysis and confirmation of hub genes

**FIGURE 5:**
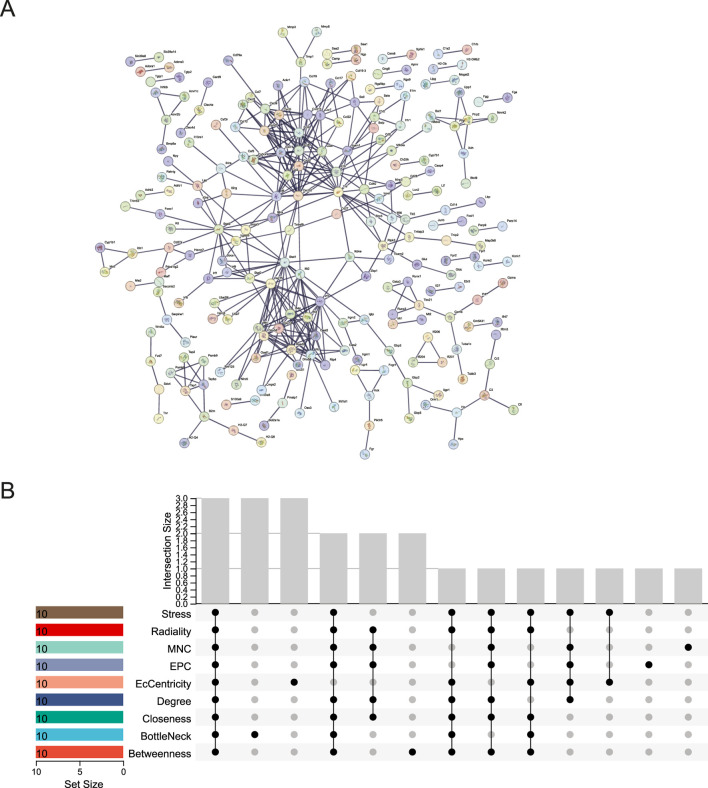
PPI network and hub gene identification. **(A)** STRING PPI Network. **(B)** UpSet Plot of Top 10 Hub Genes. UpSet plot identifying the intersection of top 10 hub genes determined by nine algorithmic analyses in CytoHubba.

We used the STRING database to create the PPI network, allowing us to investigate the interaction of DEGs in the blue and midnightblue modules. The STRING PPI network was constructed using an interaction score threshold of 0.9. The PPI network is depicted in Figure 5A. Nine algorithms (Betweenness, BottleNeck, Closeness, Degree, EcCentricity, EPC, MNC, Radiality, and Stress) were utilized with CytoHubba in Cytoscape to identify the top 10 hub genes of DEGs in the two key modules (Supplementary Table S5). The UpSetR package was employed to visualize the UpSet plot (Figure 5B). True hub genes were defined as genes that overlapped in the top 10 hub genes identified by nine different algorithms. National Center for Biotechnology Information (NCBI) Gene Summary for three identified true hub genes (Cxcl10, Il6, Stat1) are presented in Supplementary Table S6.

### 3.5 Validation and assessment of true hub genes

**FIGURE 6:**
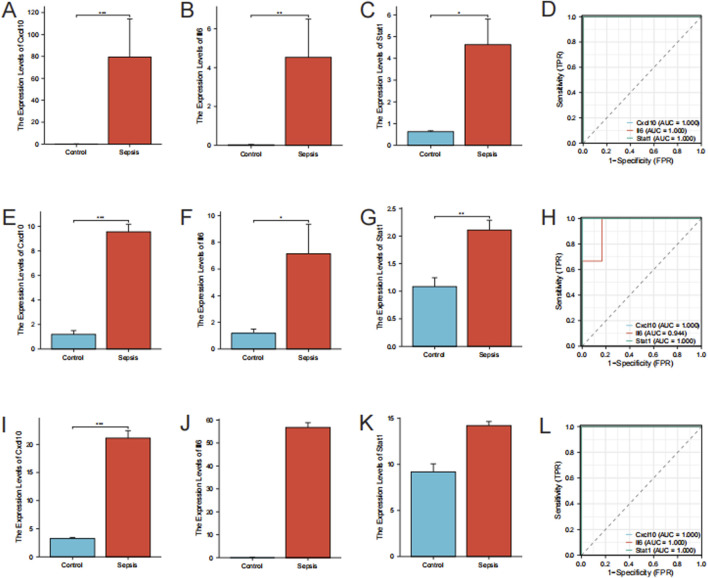
Validation of hub genes. **(A–C)** Expression Levels of Hub Genes by RNA-Seq. Bar plots showing the expression levels of Cxcl10, Il6, and Stat1 in sepsis versus control groups as determined by RNA-seq. **(D)** ROC Curves for Hub Genes from RNA-Seq. **(E–G)** RT-qPCR Validation of Hub Genes. Bar plots showing expression levels of Cxcl10, Il6, and Stat1 by RT-qPCR. Data are expressed as mean ± SD. *p < 0.05, **p < 0.01, ***p < 0.001, ns: no significance. n = 6 mice per group. **(H)** ROC Curves for Hub Genes from RT-qPCR. **(I–K)** Expression Levels of Hub Genes from the validation set GSE209706. Bar plots showing the expression levels of Cxcl10, Il6, and Stat1 in sepsis versus control groups as determined by RNA-seq. **(L)** ROC Curves for Hub Genes from the validation set GSE209706.

Cxcl10, Il6, and Stat1 showed higher levels of expression in the sepsis group than in the control group in the RNA-seq data (*P* < 0.05) (Figures 6A–C). In order to confirm the precision of these findings, we conducted RT-qPCR for authentication. The RT-qPCR results for mice gastrocnemius muscle are displayed in Figures 6E–G. In the sepsis group, there was a notable increase in the expression of Cxcl10, Il6, and Stat1, which aligns with the findings from the RNA-seq data. To validate these hub genes in an *in vitro* model, we downloaded the relevant expression profile data of the GSE209706 dataset as a validation set. In the validation set GSE209706, the sepsis (LPS-treated) group showed significantly increased levels of Cxcl10, Il6, and Stat1 compared to the control group (Figures 6I–K). ROC curve analysis was performed to evaluate the diagnostic importance of these three hub genes. In the RNA-seq dataset, Cxcl10, Il6, and Stat1 all had ROC values of 1 (Figure 6D). In the RT-qPCR results, Cxcl10 and Stat1 also had ROC values of 1, while the ROC value of Il6 was 0.94 (Figure 6H). In the validation set GSE209706, Cxcl10, Il6, and Stat1 all had ROC values of 1 (Figure 6L).

### 3.6 GeneMANIA analysis

**FIGURE 7:**
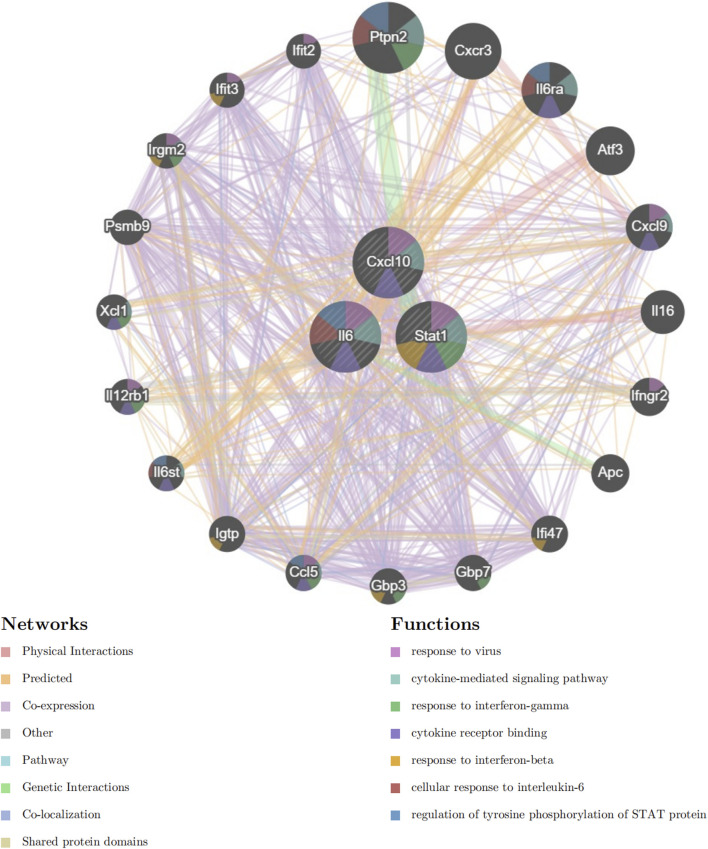
GeneMANIA Network Analysis. Network diagram generated using GeneMANIA showing the functional interactions and pathways associated with the hub genes Cxcl10, Il6, and Stat1.

Next, we included Cxcl10, Il6, and Stat1 in the GeneMANIA analysis. The GeneMANIA study shows that these processes are interconnected with virus response, signaling pathways mediated by cytokines, reaction to interferon-gamma, binding to cytokine receptors, response to interferon-beta, cellular reaction to interleukin-6, and control of STAT protein tyrosine phosphorylation (Figure 7).

### 3.7 Targeted drug prediction

**FIGURE 8:**
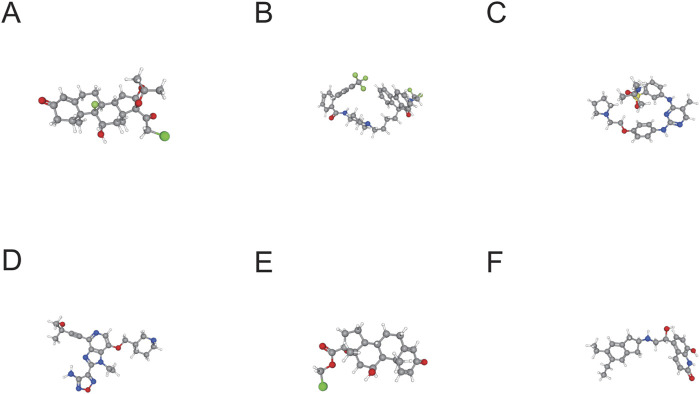
3D Structures of Candidate Small Molecule Drugs. **(A)** halcinonide. **(B)** lomitapide. **(C)** TG-101348. **(D)** GSK-690693. **(E)** loteprednol. **(F)** indacaterol.

By utilizing the CMAP database, we successfully predicted several potential therapeutic drugs for the disease. Through comparing the gene expression changes induced by the specific disease and drug treatments, we ranked the potential drugs based on their connectivity scores and selected the following top six small-molecule drug inhibitors as candidate therapies: halcinonide, lomitapide, TG-101348, GSK-690693, loteprednol, and indacaterol, as shown in Table 2. Following that, the molecular structures of the small-molecule medications were acquired from PubChem (Figure 8).

**TABLE 2:** Top six small molecule drug inhibitors.

CMAP name	Score	ID	Target	MOA
halcinonide	−0.8852	BRD-A88138582	NR3C1	Glucocorticoid receptor agonist
lomitapide	−0.8795	BRD-K92213669	MTTP|CYP2B6|CYP2C19|CYP2C8	Microsomal trigylceride transfer protein inhibitor
TG-101348	−0.8778	BRD-K12502280	JAK2|FLT3|RET|BRD4|JAK1|JAK3|TYK2	JAK inhibitor|FLT3 inhibitor
GSK-690693	−0.8744	BRD-K25325018	AKT1|AKT2|AKT3|PAK4|PAK6|PAK7|PRKCQ|PRKG1|PRKX	AKT inhibitor
loteprednol	−0.8741	BRD-K39983086	NR3C1	Glucocorticoid receptor agonist|Phospholipase inhibitor
indacaterol	−0.8708	BRD-K89208535	ADRB2|ADRB1	Adrenergic receptor agonist

## 4 Discussion

Sepsis, an inflammatory reaction throughout the body caused by infection, can result in dysfunction of various organs, such as muscle wasting and weakness, referred to as SIM (Wollersheim et al., 2014). This condition exacerbates the morbidity and mortality associated with sepsis, prolongs hospital stays, and increases healthcare costs (Hermans et al., 2014; Friedrich et al., 2015; Hermans and Van den Berghe, 2015). Despite progress in critical care, there are few successful treatments available for sepsis-induced muscle weakness, highlighting the pressing requirement for a more thorough comprehension of its molecular processes and the creation of specific therapies (Singer et al., 2016; Yoshihara et al., 2023).

In this study, we focused on elucidating the molecular mechanisms underlying SIM through comprehensive gene expression analysis and bioinformatics approaches. Our research utilized RNA sequencing and advanced data analysis techniques to uncover differentially expressed genes and construct PPI networks. Identifying true hub genes like Cxcl10, Il6, and Stat1, and confirming them with RT-qPCR, emphasizes their promise as biomarkers for diagnosis and targets for treatment. Furthermore, the prediction of potential small molecule drugs using the CMAP database offers promising avenues for developing effective treatments for SIM, potentially improving patient outcomes and reducing the burden of this debilitating condition.

Cxcl10, a chemokine, is essential in immune responses as it attracts immune cells to areas of inflammation. It is primarily induced by interferon (IFN) γ and is involved in the recruitment of T cells, NK cells, and monocytes (Norose et al., 2011). Our study identified Cxcl10 as significantly upregulated, suggesting its involvement in the inflammatory response associated with SIM. Prior research has indicated that increased amounts of Cxcl10 are linked to different inflammatory conditions, such as sepsis, where it worsens the inflammatory environment (Liu et al., 2011). The high diagnostic value of Cxcl10, as indicated by its ROC value of 1 in both RNA-seq and RT-qPCR analyses, underscores its potential as a biomarker for SIM. Furthermore, the GeneMANIA analysis linking Cxcl10 to cytokine-mediated signaling pathways and interferon responses supports its role in the immune dysregulation observed in SIM. Targeting Cxcl10 or its signaling pathways could, therefore, represent a novel therapeutic strategy for mitigating the inflammatory damage in SIM.

Il6 is a multifunctional cytokine that plays a pivotal role in the immune response, inflammation, and hematopoiesis. Various cell types, such as T cells, B cells, macrophages, and fibroblasts, generate it in reaction to infections and tissue damage (Hunter and Jones, 2015). Our research revealed that Il6 was among the main genes that were upregulated in the sepsis group, consistent with its established function as a significant player in the acute phase response (Tanaka et al., 2014). High levels of Il6 have been linked to the development of sepsis and are connected to negative results because of its inflammatory properties and capacity to trigger a cytokine storm (Schulte et al., 2013). The ROC analysis in our study demonstrated a high diagnostic value for Il6, with an ROC value of 0.94 in RT-qPCR results, indicating its potential as a biomarker for SIM. The GeneMANIA analysis further highlighted Il6’s involvement in cytokine signaling and immune response pathways, reinforcing its significance in the inflammatory processes of SIM. Therapeutic strategies aimed at modulating Il6 activity could potentially alleviate the inflammatory burden in SIM patients.

Stat1 is a transcription factor that is activated by various cytokines, including IFN-γ and IFN-α/β. Regulating the expression of genes related to immune responses, cell growth, and apoptosis is crucial for mediating the cellular response to these cytokines (Ramana et al., 2002). Our study identified Stat1 as significantly upregulated in the sepsis group, which is consistent with its role in the immune response to sepsis (Ivashkiv and Donlin, 2014). Stat1 activation leads to the transcription of numerous genes that are involved in antiviral responses and inflammation, making it a key player in the host defense mechanism (Majoros et al., 2017). The high ROC value of 1 for Stat1 in both RNA-seq and RT-qPCR analyses indicates its strong diagnostic potential for SIM. GeneMANIA analysis revealed that Stat1 is closely associated with pathways related to viral response and cytokine signaling, further supporting its involvement in the pathophysiology of SIM. Targeting Stat1 or its downstream signaling pathways could offer new therapeutic avenues for managing the inflammatory and immune responses in SIM.

Our research found that the differentially expressed genes in the blue module were strongly linked to biological processes like allergies, increased inflammation from antigen exposure, and synaptic trimming. Furthermore, the KEGG pathway analysis revealed a significant association between these genes and conditions like tuberculosis, phagosome, and alcoholic liver disease. The results indicate that the immune system’s reaction is essential in SIM. Allergic reactions and antigen-stimulated inflammatory responses are critical components of the body’s defense mechanism against infections, which aligns with the pathophysiology of sepsis, where an overwhelming immune response leads to systemic inflammation and subsequent organ dysfunction, including muscle degradation (Hotchkiss and Karl, 2003; Angus and van der Poll, 2013).

The identification of synaptic pruning as a significant biological process is particularly intriguing, as it highlights the potential involvement of neural-immune interactions in SIM. Synaptic pruning is essential for the removal of unnecessary synapses during development and in response to injury or disease, suggesting that neural pathways might be disrupted in SIM, contributing to muscle weakness and atrophy (Paolicelli and Ferretti, 2017). The association with diseases such as tuberculosis and alcoholic liver disease further underscores the complexity of the immune response in SIM. Tuberculosis and alcoholic liver disease are both characterized by chronic inflammation and immune dysregulation, which can exacerbate muscle wasting and weakness, similar to the effects observed in sepsis (Schaible and Kaufmann, 2007).

The identification of true hub genes such as Cxcl10, Il6, and Stat1, which were validated through RT-qPCR, further supports the central role of immune signaling pathways in SIM. These genetic factors play a role in the signaling of cytokines and the response to IFN-γ, which are essential for controlling immune reactions and inflammation (Kim et al., 2020). The high diagnostic value of these genes, as indicated by the ROC curve analysis, suggests their potential as biomarkers for early detection and monitoring of SIM.

Moreover, the prediction of potential therapeutic drugs such as halcinonide, lomitapide, TG-101348, GSK-690693, loteprednol, and indacaterol through the CMAP database offers promising avenues for treatment. To evaluate the translational potential of the six candidate small-molecule drugs identified via CMAP analysis, we conducted a comprehensive literature review. TG-101348 (Fedratinib), a selective JAK2 inhibitor approved for myelofibrosis (Mullally et al., 2020), has not yet been tested in sepsis but may exert therapeutic effects by modulating the JAK2/STAT1 signaling axis, a key regulator of inflammation and immune dysregulation in sepsis (Yin et al., 2024). GSK-690693, a pan-AKT inhibitor under early-phase oncology trials (Levy et al., 2009), affects pathways involved in autophagy, muscle metabolism, and cytokine regulation (Summermatter et al., 2017; Cheng et al., 2021; Ji et al., 2025), suggesting a theoretical benefit in mitigating muscle wasting. Lomitapide, an MTP inhibitor used for familial hypercholesterolemia, has been shown to modulate inflammatory cytokine expression via lipid signaling pathways (Munkhsaikhan et al., 2022), although its role in sepsis remains unexplored. Halcinonide and loteprednol, both topical corticosteroids, exhibit strong anti-inflammatory effects. While systemic corticosteroids are sometimes used in sepsis or septic shock (Annane, 2001; Sprung et al., 2011), their dual effects—anti-inflammatory versus muscle catabolic (Peris-Moreno et al., 2021)—warrant caution in SIM. Indacaterol, a long-acting β2-agonist for chronic pulmonary disease, has shown potential in reducing systemic inflammation and preserving skeletal muscle (McKeage, 2012), although it has not yet been applied to sepsis-induced muscle loss. Finally, given the pronounced upregulation of Il6 in our model, anti-IL-6 therapies such as tocilizumab appear highly relevant. Tocilizumab has been tested in sepsis-related cytokine storm settings (e.g., COVID-19), though with mixed clinical outcomes (Han et al., 2020). Nonetheless, its known efficacy in reducing IL-6–mediated muscle catabolism supports its consideration in SIM contexts. Collectively, our findings not only provide computational evidence for these candidates but also offer mechanistic rationale based on sepsis-relevant signaling pathways. These hypotheses warrant further validation *in vitro* and *in vivo*.

While our LPS-based model effectively captures key sepsis pathways like cytokine storm and mitochondrial dysfunction, its focus on Gram-negative infection necessitates future studies incorporating diverse etiologies (e.g., Gram-positive or fungal) to address clinical heterogeneity. Furthermore, transcriptomic analysis confined to the gastrocnemius muscle, though convenient and well-studied, may not represent the spectrum of muscle vulnerability, as proximal and respiratory muscles (e.g., diaphragm) often exhibit earlier or more severe impairment during sepsis (Callahan and Supinski, 2009; Chen et al., 2023); subsequent multi-muscle studies are warranted.

Despite the strengths of our study, several limitations should be acknowledged. First, the experimental validation was restricted to three hub genes (Cxcl10, Il6, and Stat1), although additional candidate genes identified through WGCNA and PPI network analysis may also possess diagnostic or therapeutic potential. Further validation of these candidates in future studies is warranted to avoid missing key biomarkers. Second, our study was conducted solely in an LPS-induced murine model, which may not fully recapitulate the pathophysiology of human sepsis-induced myopathy (SIM). Given potential interspecies differences in gene expression and immune responses, validation using clinical samples—such as muscle biopsies or blood specimens from sepsis patients—is essential to confirm the translational relevance of our findings. Third, although six candidate small-molecule drugs were identified via CMAP analysis, these results are exploratory. Extensive experimental validation—including *in vitro* screening, preclinical testing in diverse animal models, and eventual clinical trials—is required before these agents can be considered for human therapeutic use. Fourth, all mice used in this study were young males, which may limit the generalizability of the results. Biological responses to sepsis and muscle wasting are known to vary with sex and age; thus, future studies should incorporate sex-balanced and age-stratified animal cohorts. Fifth, gene expression was assessed at a single time point (18 h post-LPS injection), limiting our understanding of the dynamic changes associated with SIM progression. Longitudinal studies that monitor transcriptional changes across different disease stages are necessary to better capture the temporal evolution of this condition. Finally, a key limitation of this study is the lack of protein-level validation, which will be essential in future investigations to confirm the translational relevance of the identified hub genes at the protein level in both *in vivo* and *in vitro* models.

## 5 Conclusion

To summarize, this research thoroughly explains the molecular processes involved in SIM by discovering 1,166 genes with altered expression levels using RNA sequencing and identifying two key modules through WGCNA. Functional enrichment analyses indicated that these genes play a role in essential biological functions like immune response and inflammation. Identifying and confirming three true hub genes—Cxcl10, Il6, and Stat1—emphasizes their promise as markers for diagnosis and targets for treatment. Moreover, the prediction of six potential therapeutic drugs offers promising avenues for future clinical interventions. Additional research involving more extensive clinical data is necessary to corroborate these results and investigate the medicinal possibilities of the drugs identified.

## Data Availability

The research has uploaded all datasets analyzed to the GEO database (https://www.ncbi.nlm.nih.gov/geo/), with the data’s GEO accession number being GSE249141. The dataset will be publicly released once the article is published. All data generated or analyzed during this study are available from the corresponding author on reasonable request.
