# Supplementary material for: Identification of key modules and hub genes for sepsis-induced myopathy using weighted gene co-expression network analysis
Source: Front Genet. 2025 Jul 28;16:1607575. doi: 10.3389/fgene.2025.1607575 (PMC12336033; doi:10.3389/fgene.2025.1607575)
Supplement: Supplementary file 1 [file Table4.doc]

**Supplementary Table 4 KEGG enrichment analysis of 636 differential genes in midnightblue modules.**

| **ONTOLOGY** | **ID** | **Description** | **p-value** | **p.adjust** |
| --- | --- | --- | --- | --- |
| KEGG | mmu04060 | Cytokine-cytokine receptor interaction | 1.44463E-16 | 3.23598E-14 |
| KEGG | mmu04061 | Viral protein interaction with cytokine and cytokine receptor | 5.31543E-13 | 5.95328E-11 |
| KEGG | mmu04668 | TNF signaling pathway | 1.42503E-12 | 1.06402E-10 |
| KEGG | mmu04064 | NF-kappa B signaling pathway | 3.45228E-11 | 1.93328E-09 |
| KEGG | mmu04657 | IL-17 signaling pathway | 3.82939E-09 | 1.71557E-07 |
| KEGG | mmu05323 | Rheumatoid arthritis | 1.32915E-08 | 4.96216E-07 |
| KEGG | mmu04620 | Toll-like receptor signaling pathway | 5.87102E-07 | 1.81144E-05 |
| KEGG | mmu05164 | Influenza A | 6.46943E-07 | 1.81144E-05 |
| KEGG | mmu05162 | Measles | 1.71783E-06 | 4.27549E-05 |
| KEGG | mmu04062 | Chemokine signaling pathway | 2.77736E-06 | 6.22128E-05 |

Summary of the top 10 significant items in the KEGG enrichment analysis of 636 differential genes in midnightblue modules. KEGG: Kyoto Encyclopedia of Genes and Genomes.
