# Supplementary material for: Identification of key modules and hub genes for sepsis-induced myopathy using weighted gene co-expression network analysis
Source: Front Genet. 2025 Jul 28;16:1607575. doi: 10.3389/fgene.2025.1607575 (PMC12336033; doi:10.3389/fgene.2025.1607575)
Supplement: Supplementary file 2 [file Table6.doc]

**Additional Table 6 Introducing three true hub genes.**

| Tag | Full name | Summary |
| --- | --- | --- |
| Cxcl10 | C-X-C motif chemokine ligand 10 | Predicted to enable chemoattractant activity; chemokine receptor binding activity; and heparin binding activity. Acts upstream of or within several processes, including defense response to virus; negative regulation of myoblast differentiation; and negative regulation of myoblast fusion. Located in external side of plasma membrane and extracellular space. Is expressed in several structures, including alimentary system; axial skeleton; hemolymphoid system; and liver. Human ortholog(s) of this gene implicated in hepatitis B; middle cerebral artery infarction; and type 1 diabetes mellitus. Orthologous to human CXCL10 (C-X-C motif chemokine ligand 10). [provided by Alliance of Genome Resources, Apr 2022] |
| Il6 | interleukin 6 | This gene encodes a member of the interleukin family of cytokines that have important functions in immune response, hematopoiesis, inflammation and the acute phase response. The ectopic overexpression of the encoded protein in mice results in excessive plasma cells in circulation, leading to death. Mice lacking the encoded protein exhibit abnormalities in hepatic acute phase response, some immune mechanisms, bone resorption in response to estrogen, liver regeneration and wound healing. Alternative splicing results in multiple transcript variants encoding different isoforms. [provided by RefSeq, Sep 2015] |
| Stat1 | signal transducer and activator of transcription 1 | Enables DNA-binding transcription factor activity. Involved in activation of cysteine-type endopeptidase activity involved in apoptotic process and defense response to other organism. Acts upstream of or within several processes, including cell surface receptor signaling pathway; negative regulation of macrophage fusion; and response to exogenous dsRNA. Located in cytoplasm and nucleus. Is expressed in several structures, including alimentary system; central nervous system; genitourinary system; hemolymphoid system gland; and skeletal musculature. Used to study breast cancer and severe acute respiratory syndrome. Human ortholog(s) of this gene implicated in breast carcinoma; immunodeficiency 31A; immunodeficiency 31B; immunodeficiency 31C; and prostate adenocarcinoma. Orthologous to human STAT1 (signal transducer and activator of transcription 1). [provided by Alliance of Genome Resources, Apr 2022] |

National Center for Biotechnology Information (NCBI) Gene Summary for Cxcl10, Il6, and Stat1.
