# Supplementary material for: Identification of key modules and hub genes for sepsis-induced myopathy using weighted gene co-expression network analysis
Source: Front Genet. 2025 Jul 28;16:1607575. doi: 10.3389/fgene.2025.1607575 (PMC12336033; doi:10.3389/fgene.2025.1607575)
Supplement: Supplementary file 3 [file Table3.doc]

**Supplementary Table 3 GO Enrichment Analysis of 636 Differential Genes in midnightblue Modules.**

| **Ontology** | **ID** | **Description** | **p-value** | **p.adjust** |
| --- | --- | --- | --- | --- |
| BP | GO:0009615 | response to virus | 7.25e-28 | 2.96e-24 |
| BP | GO:0051607 | defense response to virus | 3.31e-25 | 5.17e-22 |
| BP | GO:0140546 | defense response to symbiont | 3.81e-25 | 5.17e-22 |
| BP | GO:0019221 | cytokine-mediated signaling pathway | 5.02e-21 | 5.12e-18 |
| BP | GO:0034341 | response to interferon-gamma | 2.42e-20 | 1.97e-17 |
| CC | GO:0062023 | collagen-containing extracellular matrix | 1.18e-05 | 0.0037 |
| MF | GO:0008009 | chemokine activity | 9.03e-15 | 5.3e-12 |
| MF | GO:0005125 | cytokine activity | 6.79e-12 | 1.75e-09 |
| MF | GO:0042379 | chemokine receptor binding | 8.94e-12 | 1.75e-09 |
| MF | GO:0005126 | cytokine receptor binding | 4.35e-11 | 6.39e-09 |
| MF | GO:0001664 | G protein-coupled receptor binding | 7.07e-09 | 8.3e-07 |

Summary of the top 5 significant entries in BP, CC, and MF from the GO Enrichment Analysis of 636 Differential Genes in midnightblue Modules.BP: biological processes; CC: cellular components; MF: molecular function.
