# Supplementary material for: Identification of key modules and hub genes for sepsis-induced myopathy using weighted gene co-expression network analysis
Source: Front Genet. 2025 Jul 28;16:1607575. doi: 10.3389/fgene.2025.1607575 (PMC12336033; doi:10.3389/fgene.2025.1607575)
Supplement: Supplementary file 4 [file Table1.doc]

**Supplementary Table 1 GO Enrichment Analysis of 421 Differential Genes found in Blue Modules.**

| **Ontology** | **ID** | **Description** | **p-value** | **p.adjust** |
| --- | --- | --- | --- | --- |
| BP | GO:2000425 | regulation of apoptotic cell clearance | 0.0005 | 0.0084 |
| BP | GO:0097278 | complement-dependent cytotoxicity | 0.0006 | 0.0084 |
| BP | GO:0098883 | synapse pruning | 0.0006 | 0.0084 |
| BP | GO:0002863 | positive regulation of inflammatory response to antigenic stimulus | 0.0006 | 0.0084 |
| BP | GO:0002524 | hypersensitivity | 0.0007 | 0.0084 |
| CC | GO:0035578 | azurophil granule lumen | 0.0046 | 0.0160 |
| CC | GO:0072562 | blood microparticle | 0.0075 | 0.0160 |
| CC | GO:0005766 | primary lysosome | 0.0079 | 0.0160 |
| CC | GO:0042582 | azurophil granule | 0.0079 | 0.0160 |
| CC | GO:0005775 | vacuolar lumen | 0.0089 | 0.0160 |
| MF | GO:0004866 | endopeptidase inhibitor activity | 0.0098 | 0.0187 |
| MF | GO:0030414 | peptidase inhibitor activity | 0.0102 | 0.0187 |
| MF | GO:0061135 | endopeptidase regulator activity | 0.0105 | 0.0187 |
| MF | GO:0061134 | peptidase regulator activity | 0.0125 | 0.0187 |
| MF | GO:0001664 | G protein-coupled receptor binding | 0.0156 | 0.0188 |

Summary of the top 5 important entries in BP, CC, and MF from the GO Enrichment Analysis of 421 Differential Genes found in Blue Modules. BP: biological processes; CC: cellular components; MF: molecular function.
