# Supplementary material for: Identification of key modules and hub genes for sepsis-induced myopathy using weighted gene co-expression network analysis
Source: Front Genet. 2025 Jul 28;16:1607575. doi: 10.3389/fgene.2025.1607575 (PMC12336033; doi:10.3389/fgene.2025.1607575)
Supplement: Supplementary file 5 [file Table5.doc]

**Additional Table 5 Outcome of nine Cytohubba algorithms.**

| **Rank** | **Betweenness** | **BottleNeck** | **Closeness** | **Degree** | **EcCentricity** | **EPC** | **MNC** | **Radiality** | **Stress** |
| --- | --- | --- | --- | --- | --- | --- | --- | --- | --- |
| 1 | Stat1 | Stat1 | Cxcl10 | Tnf | Socs3 | Cxcl10 | Cxcl10 | Il6 | Stat1 |
| 2 | Tnf | Cxcl10 | Stat1 | Cxcl10 | Cxcl10 | Ccl2 | Tnf | Cxcl10 | Cxcl10 |
| 3 | Cxcl10 | Tnf | Il6 | Stat1 | Il6 | Ccl5 | Ccl5 | Stat1 | Tnf |
| 4 | Stat3 | Stat3 | Tnf | Ccl5 | Stat3 | Il6 | Stat1 | Tnf | Il6 |
| 5 | Il6 | Isg15 | Il1b | Ccl2 | Ifit2 | Il1b | Ccl2 | Il1b | Stat3 |
| 6 | Isg15 | Il6 | Ccl5 | Il1b | Isg15 | Tnf | Il1b | Ccl2 | Isg15 |
| 7 | Il1b | B2m | Ccl2 | Isg15 | Stat1 | Cxcl1 | Il6 | Ccl5 | Il1b |
| 8 | Cd274 | Tbx21 | Isg15 | Il6 | Irf7 | Stat1 | Irf7 | Stat3 | Ccl2 |
| 9 | Ccl2 | Gzmb | Cxcl1 | Irf7 | Ikbke | Ccl4 | Cxcl1 | Cxcl1 | Irf7 |
| 10 | Cd40 | Il1b | Stat3 | Cxcl1 | Il6ra | Irf7 | Rsad2 | Isg15 | Ifit2 |

Lists the top 10 hub genes identified by nine Cytohubba algorithms.
