# Supplementary material for: Identification of key modules and hub genes for sepsis-induced myopathy using weighted gene co-expression network analysis
Source: Front Genet. 2025 Jul 28;16:1607575. doi: 10.3389/fgene.2025.1607575 (PMC12336033; doi:10.3389/fgene.2025.1607575)
Supplement: Supplementary file 6 [file Table2.doc]

**Supplementary Table 2 KEGG enrichment analysis of 421 differential genes in blue modules.**

| **ONTOLOGY** | **ID** | **Description** | **p-value** | **p.adjust** |
| --- | --- | --- | --- | --- |
| KEGG | hsa05134 | Legionellosis | 0.006981872 | 0.03267629 |
| KEGG | hsa05133 | Pertussis | 0.009309162 | 0.03267629 |
| KEGG | hsa05140 | Leishmaniasis | 0.009431651 | 0.03267629 |
| KEGG | hsa04610 | Complement and coagulation cascades | 0.010411563 | 0.03267629 |
| KEGG | hsa05150 | Staphylococcus aureus infection | 0.011758942 | 0.03267629 |
| KEGG | hsa05142 | Chagas disease | 0.012493876 | 0.03267629 |
| KEGG | hsa05322 | Systemic lupus erythematosus | 0.016658501 | 0.03267629 |
| KEGG | hsa04936 | Alcoholic liver disease | 0.017393435 | 0.03267629 |
| KEGG | hsa04145 | Phagosome | 0.018618324 | 0.03267629 |
| KEGG | hsa05152 | Tuberculosis | 0.022048016 | 0.03267629 |

Summary of the top 10 significant items in the KEGG enrichment analysis of 421 differential genes in blue modules.KEGG: Kyoto Encyclopedia of Genes and Genomes.
